# Perimeter confinements of basic health zones and COVID-19 incidence in Madrid, Spain

**DOI:** 10.1186/s12889-022-12626-x

**Published:** 2022-02-03

**Authors:** García-García David, Herranz-Hernandez Rafael, Rojas-Benedicto Ayelén, León-Gómez Inmaculada, Larrauri Amparo, Peñuelas Marina, Guerrero-Vadillo María, Ramis Rebeca, Gómez-Barroso Diana

**Affiliations:** 1 Consorcio de Investigación Biomédica en Red de Epidemiología y Salud Pública, CIBERESP, Madrid, Spain; 2 Centro Nacional de Epidemiología, Instituto de Salud Carlos III, Madrid, Spain; 3 Residente de Medicina Preventiva y Salud Pública, Hospital Clínico San Carlos, Madrid, Spain

**Keywords:** Covid-19, Madrid, Spain, Perimeter closures

## Abstract

**Background:**

A unique policy of perimeter closures of Basic Health Zones (small administrative health units) was implemented in the Autonomous Community of Madrid from September 21st 2020 to May 23rd 2021 to face the COVID-19 pandemic.

**Aim:**

To assess the impact of local perimeter confinements on the 14-days cumulative incidence of SARS-CoV-2 during the second wave of the pandemic in Madrid, Spain.

**Methods:**

We compare the errors in estimation of two families of mathematical models: ones that include the perimeter closures as explanatory covariables and ones that do not, in search of a significant improvement in estimation of one family over the other. We incorporate leave-one-out cross-validation, and at each step of this process we select the best model in AIC score from a family of 15 differently tuned ones.

**Results:**

The two families of models provided very similar estimations, for a 1- to 3-weeks delay in observed cumulative incidence, and also when restricting the analysis to only those Basic Health Zones that were subject to at least one closure during the time under study. In all cases the correlation between the errors yielded by both families of models was higher than 0.98 (±10^− 3^ 95% CI), and the average difference of estimated 14-days cumulative incidence was smaller than 1.49 (±0.33 95% CI).

**Conclusion:**

Our analysis suggests that the perimeter closures by Basic Health Zone did not have a significant effect on the epidemic curve in Madrid.

## Background

On March 14th 2020 the Spanish government issued the Royal Decree 463/2020 [1], declaring the state of emergency throughout the Spanish territory. This was the starting point of a battery of non-pharmaceutical interventions aimed to tackle the COVID-19 pandemic in Spain. These measures included limiting people’s mobility, closures of restaurants and businesses, capacity restrictions, cleaning protocols and others. They were uniform throughout the whole country for the duration of the state of emergency. Spain is composed of 17 Autonomous Communities, with capacity to dictate public health measures in their territories. Two months later, with the order SND/399/2020 [2] of May 9th, the easing of certain nationwide restrictions marked the start of a de-escalation protocol [3]. However, this plan was not uniform across the country, with each Autonomous Community transitioning through different phases according to its epidemiological situation.

Some policies continued to be nationwide, such as order SND/422/2020 [4], issued on May 19th, which regulated the mandatory use of masks. On June 7th, the Autonomous Communities recovered their authority to withdraw some of the measures established at the beginning of the state of emergency [5], as long as they had successfully gone through the de-escalation phases. The state of emergency ended on June 21st, with mobility being restored throughout Spain and the Autonomous Communities regaining full authority over public health measures.

From October 25th 2020 to May 9th 2021, a new state of emergency was enacted, encompassing similar non-pharmaceutical interventions to face the rise of incidence of COVID-19 [6]. In this occasion, the Autonomous Communities were able to decide what health policies were implemented in their own territories, following common guidelines given by the national government.

Basic Health Zones (BHZs) are the smallest geographical sanitary areas in Spain [7, 8]. The Autonomous Community of Madrid, in addition to other measures applicable to its entire territory, designed a system of confinements by BHZ, held from September 21st 2020 to May 23rd, 2021. These perimeter confinements were triggered by given thresholds on the registered cumulative incidence of COVID-19 cases at each of the BHZs, and were updated weekly according to the evolution of their epidemiological status. To the best of our knowledge, no other countries in Europe have enacted health policies comprising such small geographical units.

The aim of this study was to examine the effectiveness of this unique system of perimeter confinements by BHZ applied at the Autonomous Community of Madrid. We focused on the second wave of the COVID-19 pandemic (September 21st to December 20th), to isolate the effect of this policy from the start of the vaccination process in Spain, on December 27th.

## Material and methods

### Study area

**Fig. 1:**
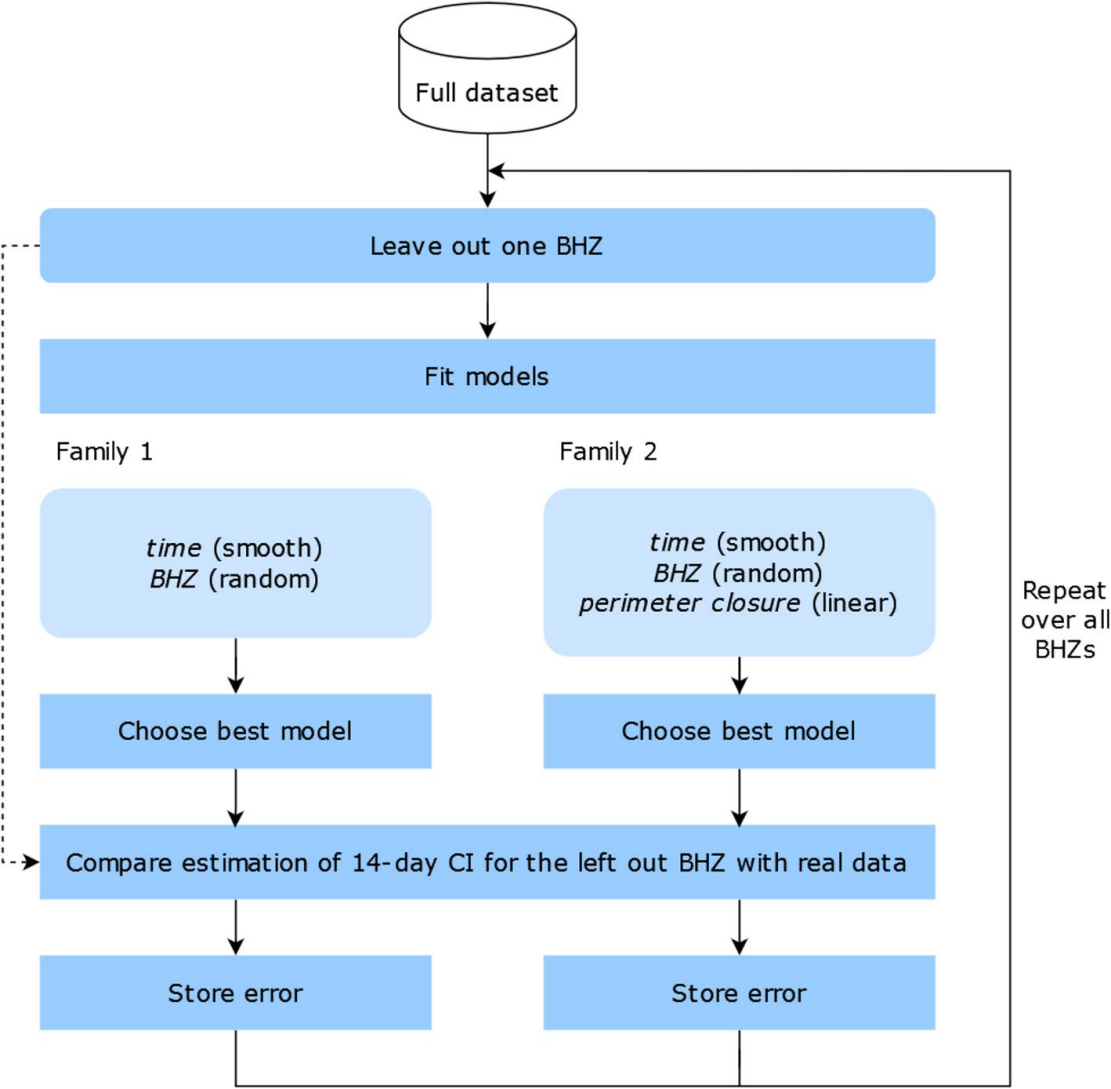
Diagrammatic description of the process followed for the statistical analysis. The type of effect assumed for each of the covariates in the models is shown in parenthesis

The Autonomous Community of Madrid is divided into 286 BHZs (Fig. 1). The total population in Madrid on January 1st 2020 was 6.663.394 inhabitants according to the National Statistics Institute of Spain official database [9].

### COVID-19 cases

The weekly 14 days cumulative incidence (CI) rates per 100,000 inhabitants at BHZ level were obtained from the open COVID-19 portal dataset of the Madrid Community Government [10]. Detailed information on the evolution of the number of COVID-19 cases across larger regions in the country is available at https://cnecovid.isciii.es/covid19/.

### Perimeter confinements of BHZs

The perimeter closures limited non-essential mobility from and to the BHZs involved, as well as non-essential activities within the BHZ. Exceptions to this restrictions included work-related mobility, access to educational centers, medical visits and other emergencies [11]. The already present restrictions at regional level were also made stricter at those BHZs with an active perimeter confinement. The range of application of these stricter measures varied during the time frame under study, but they usually involved earlier curfews and earlier mandatory closing times for restaurants and businesses (usually 0–2 h earlier times) and higher limitations in seating capacities at restaurant premises and in the number of people allowed at public gatherings (for instance, 10–20% further capacity reduction at restaurants, sports facilities and cult places, and closure of parks).

The information concerning which BHZs were confined at each point during the time of study was obtained from the Autonomous Community of Madrid’s historic public repository [11], together with the threshold that triggered the closure of a given BHZ, which changed over time (see Discussion).

While the contact patterns and general activities held at each BHZ may differ, we assumed that the number of BHZs is large enough compared to the length of the timespan under analysis (286 BHZs versus 13 weekly observations) to compensate the statistical differences between them. To further account for this effect, the BHZs were included as a random effect in the mathematical model (see Statistical analysis).

### Statistical analysis

We assessed whether the perimeter confinements of BHZs had a significant impact on the 14 days CI using Generalized Additive Models (GAMs) [12] as follows. We left out the data (cases and perimeter confinements) associated to one of the BHZs, and fitted two families of models to the resulting dataset. The models in the first family explained the 14 days CI in terms of time, and the models in the second family explained the 14 days CI in terms of time and the perimeter confinements of the BHZs. The effect of the perimeter confinements was modelled as two different covariates: one coding whether the BHZ was closed or not at each point in time, and another one coding the number of consecutive weeks the BHZ had been closed at that moment. Both families of models included the BHZ locations as a random effect as well.

Within each of the two families of models, several parameters were tuned differently in search for the best fit possible, comprising 15 models in total (we used thin plate splines, Duchon splines and cubic regression splines, and either 8,12,16,20 or 24 basis functions for the smooth temporal component). We chose the best model in AIC score from each of the families, and used these to estimate the 14 days CI of the BHZ that was left out. We then stored the absolute values of the errors in estimation (obtained as the absolute value of the difference between the best models’ output and the observed incidence). This procedure was repeated once for every BHZ, so that every time a different BHZ was left out of the fitting process, resulting in two sets of errors (each one associated to each of the families of models). Figure 1 shows a schematic description of this process.

Finally, we searched for statistically significant differences in these two sets of errors with a Pearson’s product-moment correlation test and a paired TOST [13], looking for possible consistent improvements in estimation for one family of models over the other. We performed this procedure with a time lag of 1 to 3 weeks in the observed CI, as this has been identified as the relevant time delay for the introduction of COVID-19 related non-pharmaceutical interventions to take effect [14]. Additionally, in order to magnify the possible differences, we repeated the whole process restricting the dataset to only those BHZs that were confined for at least 1 week during the time under study.

## Results

Basic Health Zones are administrative health areas of the territory, with 22,752 inhabitants in average (min = 2615, max = 59,932) and spanning 28km^2^ in average (min = 0.19 km^2^, max = 519 km^2^). Large urban territories usually enclose several BHZs, while in rural areas a single BHZ may comprise several municipalities.

**Fig. 2:**
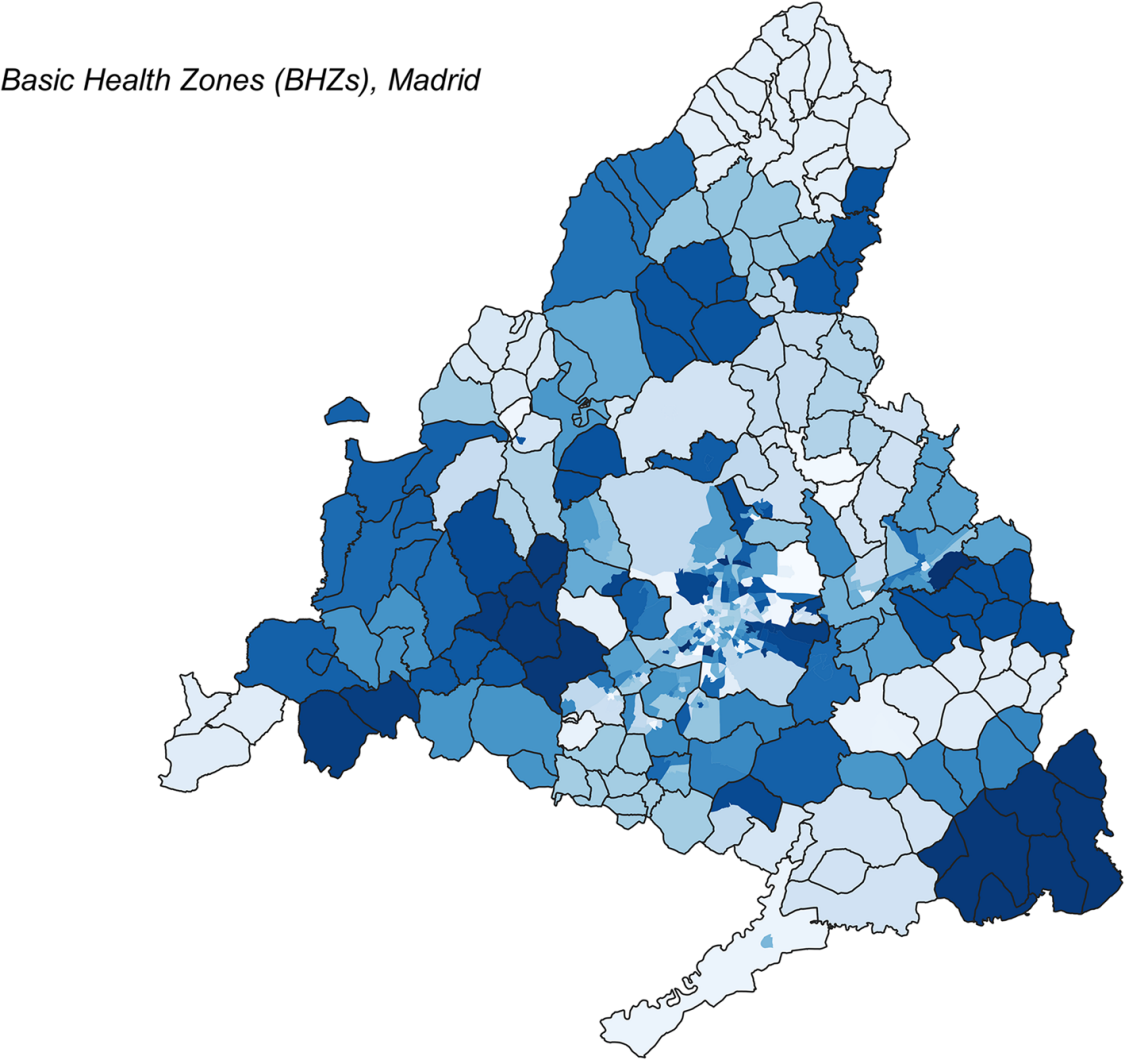
Spatial distribution of the Basic Health Zones (BHZs) in Autonomous Community of Madrid (in blue), and municipalities (black outline). Different shades of blue are used only for improved legibility

**Fig. 3:**
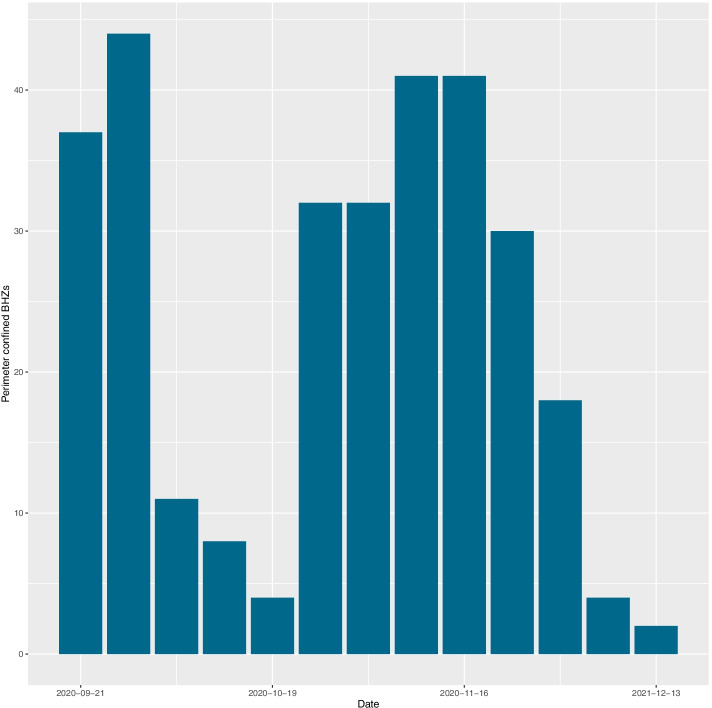
Total number of perimeter confined BHZs by date

Figure 2 shows the spatial distribution of the 286 BHZs in Madrid, and in the site https://coviddifusion.isciii.es/PerimBHZs/ we display the evolution in space and time of closures together with the cumulative incidence. The number of closed zones was heterogeneous over the time under study, taking into account the different thresholds used and the evolution of the epidemic (see Fig. 3). A total of 81 BHZs were confined for at least 1 week during the time period under study with an average of 0.29 closed weeks per BHZ among these from the total of 13 weekly observations per BHZ. During this time span, the average 14-days CI per BHZ was 387.15 cases, rising to 476.45 when considering only those BHZs that have been perimeter confined for at least 1 week during this period, with a maximum recorded incidence of 1883.1.

Table 1 shows the results of a Pearson’s product-moment correlation test and a paired TOST test for equivalence for the two sets of errors obtained with the statistical models. We find very high correlations and negligible differences in means between the two sets. These results were consistent when considering either 1, 2 or 3 weeks of lag in the observed CI, and also when restricting the analysis to only those BHZs that were closed for at least 1 week.

**Table 1:** Correlation and difference in means tests for the two sets of errors obtained from the analysis

Statistical test	Weeks of lag	All BHZs	BHZs that have been closed at least once
Pearson’s product-moment correlation test	1	Correlation: 0.99 (±10^−3^ 95% CI)	Correlation: 0.98 (±10^−3^ 95% CI)
	2	Correlation: 0.99 (±10^−4^ 95% CI)	Correlation: 0.99 (±10^−4^ 95% CI)
	3	Correlation: 0.99 (±10^−5^ 95% CI)	Correlation: 0.99 (±10^−5^ 95% CI)
Paired TOST	1	Mean of differences: 1.49 (±0.33 95% CI)	Mean of differences: 1.4 (±0.87 95% CI)
	2	Mean of differences: 0.41 (±0.13 95% CI)	Mean of differences: 0.17 (±0.33 95% CI)
	3	Mean of differences: 0.65 (±0.12 95% CI)	Mean of differences: 0.19 (±0.11 95% CI)

## Discussion

This study evaluates the impact of the local perimeter confinements implemented during the second wave of COVID-19 in Madrid. This Autonomous Community adopted a special model of public health measures based on perimeter closures by BHZ depending of the epidemiological situation across time. Our mathematical models showed no statistical differences in cumulative incidence for BHZs with and without perimeter closures.

In Spain, the universal use of masks (indoors and outdoors) is mandatory throughout the whole territory since May 19th 2020 [4]. On October 25th, the second state of emergency started and additional public health measures were implemented, with a range of normatives that varied across the Autonomous Communities. These included curfews (set at 23:00 in Madrid for the time under study), limited seating capacities at restaurant premises and gathering limitations (maximum of 6 people in outdoors restaurant facilities and in-house meetings, and 4 people in indoors restaurant facilities in Madrid), and other general restrictions [15]. Prior to the state of emergency, the perimeter confinements by BHZ were also activated, in contrast to the municipality-level closures adopted at other Autonomous Communities (Galicia [16], Cantabria [17], among others).

We assessed whether the perimeter confinements of BHZs had a significant influence on the evolution of the epidemic curve by modeling them as explanatory covariables in several mathematical models. We found that the estimations provided by the models that included the perimeter confinements as an explanatory variable and those that did not were statistically very similar, indicating that the perimeter confinements did not have a significant impact on the 14 days accumulated CI.

Several factors limit the effectiveness of the BHZ closures system. For example, due to the high permeability between neighboring BHZs and associated difficulty in the evaluation of the citizens’ compliance to the measure, it has not been possible to determine if the policy was implemented effectively. In addition, a low risk perception towards the COVID-19 pandemic has been identified in the Spanish population during the time period under study [18], which could have been resulted in a decreased adherence to the policy [19, 20].

While local mobility restrictions are effective in a theoretical modelling framework [21, 22], evidence suggests that an informed and coordinated approach is required for the effective implementation of such a response measure [23]. Being a rare policy, few studies that focus on the effect of such selective confinements of such small units as BHZs are available. Fotán-Vela et al. [24] also analyze the case of the BHZ closures in Madrid. Their analysis shows that the decrease in the epidemic curve in Madrid started before the impact of the perimeter closures could be reflected. Other than Madrid, the only other context were a similar policy has been adopted is Chile, to our best knowledge. Cuadrado et al. [25] and Li et al. [26] study the local lockdowns active during the first wave of the COVID-19 pandemic in this country, obtaining, respectively, a reduction in effective reproductive number (with a wide confidence interval, nevertheless), and a highly variable effectiveness of the policy (depending on duration of intervention and spillover effect from neighboring areas).

### Limitations of the analysis

The average BHZ is an epidemiologically small unit, both in terms of population (22.750 inhabitants) and area (28 km^2^). Because of this, the usual joint point methods for trend analysis will presumably not reveal meaningful conclusions at local BHZ level, lacking statistical significance. This is the case as well for trend analysis on models that incorporate information from all the BHZs, due to the asynchronicity in the implementation of the perimeter confinements among each of the BHZs. For the very same reason, precise estimations are not expected to be obtained from models fitted to this data. We thus chose to employ the present approach, sensible to general tendencies in models that have been adjusted differently, and focused on statistical assessments rather than in accurate predictions. GAM models are expected to capture a greater influence of the additional explanatory variables included than trend analysis models [12], and we incorporated higher significance by a leave-one-out cross-validation process over the 286 BHZs that involves the choice of the best of 15 models in each step.

An additional confounding effect is due to the fact that perimeter confinements (and COVID-19 related restrictions in general) have been introduced on an a posteriori basis. That is, restrictions are activated as a response to the increment of the 14 days CI, and therefore there is a natural correlation between BHZs with high CI and perimeter confined BHZs. Again, an approach that does not focus on assessing the explicit, precise impact of these restrictions and rather on its statistical effect is thus preferred, as misleading associations may be inferred otherwise.

Finally, the epidemiological threshold triggering the closures changed during the study period. On September 21st, weekly BHZ perimeter confinements were activated at those BHZs where the 14 days cumulative incidence surpassed the 1.000 cases per 100.000 inhabitants. This threshold was decreased to 750 cases on October 12th, 500 cases on October 26th, and 400 cases on November 23rd [11]. We have not included the possible effect of this variation in our analysis, as we focused on the effect of the actual perimeter confinements and not on their dependence to the current epidemiological status.

## Conclusion

Our analysis shows that the perimeter closures by BHZ do not have a significant effect on the epidemic curve in Madrid either 1, 2 or 3 weeks after their activation.

## Data Availability

The datasets generated and/or analysed during the current study are available in the public repository of the Autonomous Community of Madrid: https://www.comunidad.madrid/servicios/salud/comunicados-covid-19-normativa-notas-prensa#repositorio-historico-medidas-adoptadas-crisis-sanitariaria. https://datos.comunidad.madrid/catalogo/dataset/covid19_tia_zonas_basicas_salud.
